# Specific *TP53 *mutations predict aggressive phenotype in head and neck squamous cell carcinoma: a retrospective archival study

**DOI:** 10.1186/1758-3284-3-20

**Published:** 2011-04-22

**Authors:** Jenni K Peltonen, Kirsi H Vähäkangas, Henni M Helppi, Risto Bloigu, Paavo Pääkkö, Taina Turpeenniemi-Hujanen

**Affiliations:** 1 Department of Oncology and Radiotherapy, Oulu University Hospital, University of Oulu, Oulu, Finland; 2 Department of Pharmacology and Toxicology, University of Oulu, Finland; 3 Faculty of Health Sciences, University of Eastern Finland, Kuopio, Finland; 4 Medical Informatics Group, University of Oulu, Finland; 5 Institution of Diagnostic, Department of Pathology, University of Oulu, Oulu University Hospital, Finland

## Abstract

**Background:**

Head and neck squamous cell carcinoma (HNSCC) is the sixth most common malignancy in the world in developed countries. Despite the intense research in the area of squamous cell carcinomas of head and neck (HNSCC), long-term survival rate has not changed significantly in this malignancy during recent decades.

**Methods:**

In this study, we focused on *TP53 *mutations in specific regions, including DNA-binding surface, to determine whether mutations at specific locations of *TP53 *could be used to help in setting up prognosis and response to therapy of head and neck squamous cell carcinoma patients. We analysed *TP53 *mutations in 46 HNSCC by PCR-SSCP and sequencing and characterized how different *TP53 *mutations affect the patient outcome.

**Results:**

Tumours containing *TP53 *mutations in DNA-binding regions (L2, L3 and LSH motif) had a significantly poorer prognosis and response to radiotherapy than tumours outside those regions. Disease-specific 5-year survival of patients with *TP53 *mutations affecting DNA contacts was 43.5% while it was 77.8% (p < 0.05) in patients with *TP53 *mutations in other residues not involved in DNA contact. Moreover, nodal metastasis were more prevalent (although not statistically significantly) with *TP53 *mutations in DNA-binding surface regions. We noticed that the patients with *TP53 *mutations in L3/LSH motifs had a significantly poorer response (11.4% responding) to radiation than the patients with a wild type p53 (48.6%) or *TP53 *mutations outside the DNA-binding regions (40%) (p < 0.05).

**Conclusions:**

These data indicate that a *TP53 *mutation in L2, L3 or LSH is worth pursuing as a marker for predicting prognosis and response to radiation among HNSCC patients.

## Introduction

Head and neck squamous cell carcinoma (HNSCC) is one of the 10 most frequent malignancy in the world and more than 500 000 new cases are reported annually [1]. Despite the intense research in the area of squamous cell carcinomas of head and neck (HNSCC), the long-term survival rate has not changed significantly in this malignancy during recent decades [2]. The initial treatment approaches for a patient with HNSCC include most often radiotherapy added to primary surgery one way or another or as a definitive treatment of inoperable disease [3]. In the past decade, the role of organ-preservation protocols, with combined chemoradiation and surgery for salvage, has increased. These protocols are particularly effective for patients with moderately advanced cancers of the larynx and pharynx who are less than 70 years old and have a good performance status [2]. Also technical improvements have decreased late radiotherapy side-effects. Recently Nutting and co-workers (2011) compared conventional radiotherapy to parotid-sparing intensity-modulated radiotherapy (IMRT) in patients with pharyngeal squamous cell carcinoma and noticed significantly lower late side-effect rate but did not report any survival advantage [4]. Anti-EGFR mAb cetuximab has shown promising antitumour activity with tolerable toxicity profile but the optimal combinations and schedule is still to be found [5].

The abrogation of p53 function through the mutation of its gene, *TP53 *[6], the loss of heterozygosity of *TP53 *[7] or interaction with viral proteins [8], is one of the most common molecular alterations in squamous-cell carcinoma of the head and neck [9]. The *TP53 *tumour suppressor gene in chromosome 17p13.1 encodes the p53 protein, which functions primarily as a multi-target transcription factor. The p53 protein is known to be involved in various cellular functions including cell-cycle regulation, senescence, apoptosis, repair of DNA damage caused by genotoxic agents, angiogenesis, and regulation of oxidative stress and glucose metabolism [10-13]. Loss of the p53 function allows proliferation of the cells with a DNA-damage and promotes neoplasia in transgenic p53 null mice [14]. *TP53 *gene alterations are commonly found in head and neck cancers, and most of the published mutations affect the p53-DNA interactions, resulting in a partial or complete loss of transactivation functions [15]. *TP53 *differs from other tumour suppressor genes in its mode of inactivation. While most tumour suppressor genes are inactivated by mutations leading to absence of protein synthesis or production of a truncated protein, more than 80% of *TP53 *alterations are missense mutations that lead to the synthesis of a stable full-length protein [15]. The location of the resulting amino-acid substitution is usually within the central DNA-binding domain of the p53, resulting in a loss of DNA-binding activity with consequent failure to transcriptionally activate target genes [16].

In the past 10 years, systematic data from the functional assays have been generated and integrated in the *TP53 *database managed by the International Agency for Research on Cancer (IARC) (http://www-p53.iarc.fr/) [17]. Most mutant p53 proteins have lost their DNA-binding activity, leading to a loss of their growth inhibiting and apoptotic properties. The role of p53 as a prognostic marker of squamous cell carcinoma of the head and neck is controversial. The possible reasons for this include small numbers of patients studied, insufficient clinical follow-up, variable laboratory techniques used, or analysis of *TP53 *data based on insufficient mutation characterization [15,18].

In the present study, we pursue the clinical importance of *TP53 *mutation types in head and neck cancer. We analyzed *TP53 *mutations in 46 patients with head and neck squamous cell carcinomas (HNSCC) by polymerase chain reaction-single-strand conformation polymorphism (PCR-SSCP) and sequencing as earlier described [19]. The aim of this study was to examine whether specific *TP53 *mutation types were associated with the clinical outcome of head and neck cancer, such as lymph node metastasis, prognosis and therapy of the patients.

## Materials and methods

### Patients and tumours

In this same population, we have investigated earlier p53 aberrations in association with environmental exposures [19]. This study population consists of 46 patients treated for primary head and neck squamous cell carcinoma enrolled as patients in the University Hospital of Oulu, Finland between the years 1994 and 1996. The patients were recruited to the study before any treatment was started. We wanted a long follow-up time to clarify long-time survival differences between specific *TP53 *mutations. Clinico-pathological variables are given in Table 1. The age of the patients varied greatly (28-85 years). The stage of the disease, tumour size and lymph node involvement were determined according to the International Union Against Cancer TNM classification (1997) [20]. The histological grade of the tumours were reviewed and classified according to the World Health Organization classification of head and neck tumours [21]. The treatment was carried out according to the stage of the disease and the routine clinical treatment protocol (Table 2). 33 patients received 50 Gy or more radiotherapy as part of their treatment with conventional fractionation, five patients were treated with radical surgical operation without other treatments, and five patients received only radiotherapy (50-64 Gy). No patients were treated with adjuvant chemotherapy. Three of the patients had an advanced carcinoma and received only palliative treatment.

**Table 1:** Clinicopathological variables

Patient characteristics	n
All patients	46
Sex	
Male	31
Female	15
Age, years	
≤39	3
40-65	23
≥66	20
Anatomical location	
Oral cavity	14
Larynx	24
Pharynx	6
Others	2
Grade	
Grade 1	10
Grade 2	29
Grade 3	7
TNM classification	
T_1-2_	29
T_3-4_	17
N_0_	23
N_+_	23
Stage	
I	6
II	9
III	20
IV	11

Earlier published in Peltonen *et al. Head & Neck Oncology *2010 **2**:36 [19]

**Table 2:** Features of the primary tumours regarding primary treatment regimen, follow up time and cause of death

Tumour	Age	Disease site	Stage	TP53 gene status	Surgery	Radiation- therapy (radical)	Disease free in months	Overall survival in months	Death related to HNSCC
1	65	larynx	2	wild type	Yes	Yes	166	166	No

2	61	larynx	3	wild type	Yes	Yes	145	145	No

3	56	pharynx	3	mutation outside L2/L3/LSH motif	Yes	Yes	156	156	No

4	65	larynx	3	mutation outside L2/L3/LSH motif	Yes	Yes	121	121	No

5	48	pharynx	3	wild type	Yes	Yes		74	Yes

7	48	oral cavity	3	wild type	Yes	Yes	158	158	No

8	75	oral cavity	2	wild type	Yes	Yes	6	15	Yes

9	28	oral cavity	3	mutation outside L2/L3/LSH motif	Yes	Yes	155	155	No

10	58	oral cavity	3	wild type	Yes	Yes	11	8	Yes

12	78	oral cavity	2	mutation in L2/L3/LSH motif	Yes	No	7	40	Yes

13	83	larynx	2	wild-type	Yes	Yes	51	51	No

14	45	larynx	1	wild type	Yes	Yes	122	122	No

16	85	pharynx	3	mutation in L2/L3/LSH motif	Yes	Yes	32	32	No

17	74	oral cavity	3	wild type	Yes	No	3	12	Yes

18	72	larynx	1	mutation outside L2/L3/LSH motif	Yes	Yes	164	164	No

19	57	larynx	4	mutation outside L2/L3/LSH motif	Yes	Yes	24	24	No

20	62	oral cavity	3	mutation in L2/L3/LSH motif	No	only palliative	0	1	Yes

26	68	larynx	4	wild type	Yes	Yes	9	18	Yes

28	65	oral cavity	4	mutation in L2/L3/LSH motif	No	only palliative	6	22	Yes

29	33	oral cavity	3	mutation outside L2/L3/LSH motif	Yes	Yes	157	157	No

31	71	oral cavity	1	mutation in L2/L3/LSH motif	Yes	No	20	84	Yes

32	39	oral cavity	3	mutation in L2/L3/LSH motif	Yes	Yes	37	40	Yes

39	68	larynx	4	wild type p53	Yes	Yes	11	19	Yes

41	53	nose	2	wild type p53	Yes	Yes	7	18	Yes

43	74	larynx	2	mutation in L2/L3/LSH motif	No	Yes	154	154	No

45	70	pharynx	3	wild type p53	Yes	Yes	142	142	No

46	62	larynx	1	mutation outside L2/L3/LSH motif	Yes	Yes	135	135	No

49	62	oral cavity	1	mutation in SSCP	Yes	No	60	67	Yes

50	49	larynx	3	wild type p53	Yes	Yes	122	122	No

51	63	larynx	2	mutation outside L2/L3/LSH motif	Yes	Yes	109	109	No

52	63	larynx	4	wild type p53	Yes	Yes	20	30	Yes

53	74	oral cavity	4	mutation in L2/L3/LSH motif	Yes	No	35	37	Yes

54	42	larynx	3	mutation outside L2/L3/LSH motif	Yes	Yes	144	144	No

55	71	larynx	1	wild type p53	Yes	Yes	145	145	No

56	59	larynx	4	mutation in L2/L3/LSH motif	Yes	Yes	155	155	Yes

58	74	pharynx	4	mutation outside L2/L3/LSH motif	No	Yes	29	32	Yes

59	56	oral cavity	3	wild type p53	Yes	Yes	163	163	No

60	69	larynx	4	mutation in L2/L3/LSH motif	Yes	Yes	22	22	No

61	63	larynx	3	mutation in L2/L3/LSH motif	No	Yes	15	15	No

62	74	larynx	2	wild type p53	Yes	Yes	25	25	No

63	69	larynx	3	mutation in L2/L3/LSH motif	Yes	No	5	7	Yes

64	56	larynx	3	mutation in L2/L3/LSH motif	Yes	Yes	151	151	No

65	75	larynx	3	wild type p53	Yes	Yes	20	25	Yes

68	76	pharynx	4	SSCP positivity	No	only palliative	0	4	Yes

69	65	larynx	2	mutation outside L2/L3/LSH motif	No	Yes	12	43	Yes

70	68	sinonasal	4	wild type p53	Yes	Yes	160	160	No

### Ethical aspects

The study design was approved by the local Ethics Committee of the Medical Faculty at the University of Oulu, Finland and a written informed consent was obtained from all patients entering the study.

### p53 immunohistochemistry

Paraffin embedded sections (4 μm) from the primary tumours of head and neck carcinomas were stained using the avidin-biotin-immunoperoxidase technique. Paraffin sections were incubated at 37°C for at least 4 hours, dewaxed (Histo- Clear^®^, National Diagnostic, Atlanta, GA, USA) and hydrated. Endogenous peroxidase activity was blocked by incubating the slides in 0.1% hydrogen peroxidase/methanol for 20 min and non-specific binding was blocked with 10% goat serum for 15 min. A mouse monoclonal antibody (DO-7, Novocastra Laboratories Ltd., Newcastle upon Tyne, UK) for p53 was used as a primary antibody (1:300) mixed with 1% bovine serum albumin. The antibody recognizes both wild type and mutant forms of human p53 and the epitope is located between amino acid residues 19 and 26. The specimens were incubated for 60 minutes at room temperature in a humidified chamber after which the immunohistochemical staining was continued using Histostain-bulk kit^® ^(Zymed, San Francisco, CA, USA) according to the manufacturer's protocol. Biotinylated antimouse IgG was used as a secondary antibody and the peroxidase was introduced as a streptavidin conjugate. The slides were washed thoroughly with phosphate-buffered saline between all stages of the procedure. The antibody reaction was visualised by using a fresh substrate solution containing aminoethyl carbazol substrate kit (AEC-kit^®^, Zymed, San Francisco, CA, USA). The sections were counterstained with hematoxylin, dehydrated and mounted in glycerol-vinyl-alcohol (GVA mount^®^; Zymed). For negative controls the primary antibody for p53 was replaced with mouse non-immuno IgG and each set of staining always included a separate known positive control sample.

### Evaluation of the p53 immunostaining

The slides were analysed separately by two independent observers blinded from the clinical data. The immunoreactivity in the malignant cells in each section was graded according to the extent and amount of the positive staining from 0 to ++++. The immunoreactivity in the malignant cells in each section was graded according to the number of positively staining nuclei: < 1% nuclei with a negative reaction, 1% > ≤ 5% as +, >6% ≤10% as ++ >11% ≤40% as +++ and > 40% as ++++.

### *TP53 *mutation analysis strategy

We analyzed TP53 mutations in exons 5-8, where most of the mutations occur in human tumours (http://www-p53.iarc.fr/) by using a temperature-controlled non-radioactive single-strand conformation polymorphism (SSCP) analysis [22,23]. A sample was judged to be positive for a *TP53 *mutation in SSCP only if two independent amplified PCR products contained similar shifted band patterns. Artefacts due to formalin-fixation or the infidelity of the polymerase-enzyme in PCR were ruled out by repeating the analysis. Further characterisation of *TP53 *mutations was carried out by semi-automatic sequencing: the PCR amplified samples were sequenced with an ABI PRISM 3100 sequencer and BigDye Terminator Sequencing Kit (Applied Biosystems, Foster City, CA).

### Amplification of p53 exons by PCR

Exons 5-8 of the *TP53 *gene were separately amplified by PCR using two sets of intron primers, the second set internal to the first, i.e. nested primers [24]. The following reagents and concentrations were used in the amplifications: For the primary PCR, formalin-fixed paraffin-embedded tumour DNA were used as a template in a total volume of 100 μl with 3.0 U of Dynazyme DNA polymerase (Finnzymes, Finland), in a buffer containing 10 mM Tris-HCl (pH 8.8), 1.5mM MgCl_2_, 50mM KCl and 0.1% Triton X-100, 20-40 pmol of each primer and 300 μl of dNTPs (Pharmacia Biotech, Finland). The amplification was carried out by 35 cycles including denaturation at 94°C for 1 min, annealing at 60°C for 1 min and elongation at 78°C for 30 seconds. Secondary PCR was done in a total volume of 200 μl and 5 μl of amplified DNA from the primary PCR was used as a template. To check for possible contamination, the first and the last reactions in each PCR series were controls with no template in the reaction. If a band appeared indicating contamination, the whole series of PCR reactions was disposed of, and the analysis redone. The amplified products were purified by agarose gel electrophoresis, as described earlier [25].

### Single-strand conformation polymorphism (SSCP) analysis and sequencing

A non-radioactive PCR-SSCP method was used as previously described [19,25] with the Pharmacia PhastSystem^® ^semi-dry electrophoresis equipment. Two different temperatures (4°C and 20°C) were used to obtain good efficiency. Several studies have shown that the optimization of SSCP conditions is essential for analytical sensitivity and efficiency (for review see) [26]. Both negative and positive controls were included in each run to ensure the quality of the run. As a negative control, gel-purified, amplified normal *TP53 *DNA was used. The controls were confirmed to be negative by selecting samples where bands looked the same in SSCP as in former controls, and by additionally sequencing them to confirm the wild-type. As a positive control, DNA was amplified using artificially mutated primers [22]. The gels were stained with silver staining kit (Pharmacia Biotech, Finland) according to the instructions from the manufacturer.

### Statistical analysis

The correlations of tumour stage, TNM classification, histological grade, gender, age, and primary anatomical site were analyzed separately according to the *TP53 *gene mutations and p53 immunoreactivity. Disease-free time (DFT), disease-specific survival (DSS) and overall survival (OS) were analysed for the HNSCC patients with respect to p53 protein staining and *TP53 *mutations, using the Kaplan-Meier method and Fisher's exact test. Survival was defined as the time from the date of diagnosis to death due to the cancer or the date of the last follow-up visit. Disease-free time was defined as the time from diagnosis of the cancer to recurrence or death to cancer. Overall survival and disease-specific survival time was calculated from the date of diagnosis until death. The patients were censored on the date of the last follow-up examination or the date of the collection of clinical data, 24/11/2009. Probability values < 0.05 were considered to be statistically significant. All statistical analyses were performed using the SPSS software system (SPSS for Windows, version 16.0, Chicago, IL).

## Results

### General outcome

The mean follow-up of patients was 80 months (range 1-166 mo). At the time of analysis (11/2009), 39% (18 of 46) of the patients had relapsed and died for cancer. Disease-specific 5-year survival was 60.7% and overall survival 52.2% for the whole series. Overall 5-year survival was 41.2% in the group of the patients with a T3 or T4 tumour.

### Mutation analysis of *TP53 *gene

Judging by SSCP the *TP53 *gene was mutated in a total of 26 primary tumours (57%) in the 46 HNSCC patients [19]. Overall, 39 *TP53 *mutations were identified in these 26 tumours and sequencing was possible in 23 tumour samples. The features of the *TP53 *mutations are summarized in Table 2. When taking into account the functional and structural domains of p53 as described in the IARC *TP53 *mutation database (http://www-p53.iarc.fr/) [17]: 22% (8/36) of the mutations affect the L2 domain (between codons 164 and 194), which is needed for the correct folding and stabilization of the central part of the protein, 11% (4/36) affect the LSH (loop-sheet-helix) motif (codons 119-135 and 272-287), and 8% (3/36) affect the L3 domain (between codons 237-250), directly involved in the interaction between the protein and DNA. Details of the characterization of the mutations have been published separately [19].

### Comparison of p53 aberrations with clinical characteristics

The p53 expression levels, as well as the presence of *TP53 *mutations were compared with the clinical data. The p53 aberrations have been published previously and they do not associate with histological grade, TNM classification, the stage of the disease or the age of the patients [19]. Furthermore, even when comparing the specific *TP53 *mutations (e.g. *TP53 *mutations in DNA-binding domains and *TP53 *mutations outside those regions) and stage, TNM-classification or tumour histological grade, there was no differences between those two groups.

### Disease recurrence and survival of HNSCC patients related to p53 alterations

**Figure 1:**
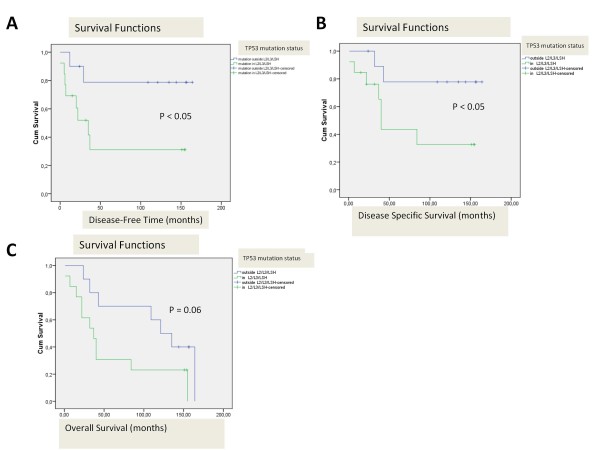
***TP53 *mutation status and survival in patients with *TP53 *mutations in DNA-binding domains and in patients with mutations outside the DNA-binding domains**. A. Disease-free time. B. Disease-specific survival. C. Overall survival.

The p53 levels as well as the presence of *TP53 *mutations were compared with the clinical data. There was no difference in survival or disease recurrence in patients when all *TP53 *mutations or different grades of p53 expression were taken on account. Patients affected by tumours with *TP53 *mutations in the DNA-binding domains L2, L3 or LSH, however, had a shorter survival. This trend was also seen when disease-specific death was compared: Patients who had *TP53 *mutations in L2, L3 and LSH motifs died of cancer more often (7 out of 9 cases, 77.7%), than patients with a wild-type p53 in their tumour (8 out of 19, 42.1%) or patients with tumours presenting a mutation outside L2/L3/LSH domain (2 out of 8 cases, 25%). We also noticed that patients with *TP53 *mutations in L3/LSH motif had more node metastases (83%) compared to patients with a wild-type tumour (50%). However, this difference was not statistically significant (p = 0.2). An interesting finding was that the patients with *TP53 *mutations in DNA binding surface region had a higher number of late residives than the patients with a wild-type p53 or patients with *TP53 *mutations outside the functional domains of p53. The patients with *TP53 *mutations in motifs L2/L3/LSH relapsed more often (eight relapses of 13 cases vs. two relapses of 10 cases) and their disease free survival time was shorter than in other cases (5-yrs rate 20% vs. 61.5%, p < 0.05, Figure 1A).

Interestingly, patients that had mutations outside the functional domains of *TP53 *in the primary tumours survived longer than the patients with a wild-type p53 in their tumour. The group of 13 patients with *TP53 *mutations affecting the DNA-binding domain had a statistically significantly shorter disease-specific survival time (5-yrs survival 43.5% vs. 77.8%, p < 0.05, Figure 1B) and a trend for a shorter overall survival time at the time for analysis 11/2009, p = 0.06 (5-yrs rate 30.8% vs. 70.0%, p < 0.05, Figure 1C) than patients with mutations outside the DNA-binding region. Strong p53 staining was associated with a better disease free time, but no correlation was found between the overall survival and p53 staining.

### *TP53 *mutations and cancer treatment

**Figure 2:**
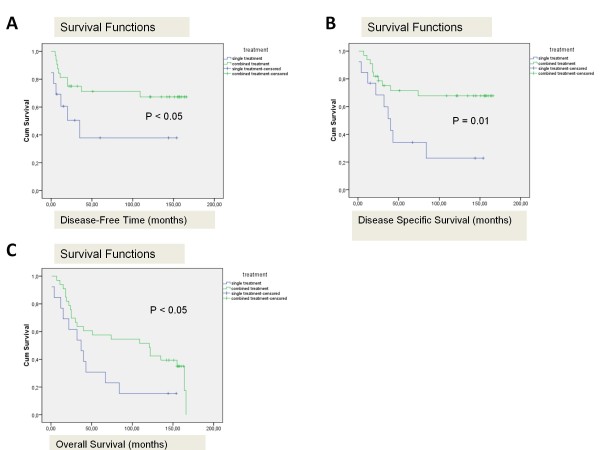
**Cancer treatment and prognosis**. Patients treated with combined treatment had a better disease-free time (A), disease-specific-survival (B), as well as overall survival (C).

The patients with *TP53 *mutations in L3/LSH motif had significantly poorer response to radiation than patients with a wild type p53 or *TP53 *mutations outside the DNA-binding regions (11.4% vs. 48.6% vs.40% p < 0.05, Pearson Chi-Square Test). Moreover, patients with *TP53 *mutations in important DNA-binding motifs L2, L3, or LSH received less combined treatment (surgery and radiotherapy), compared to patients with a *TP53 *mutation outside the L2/L3/LSH motif or a wild-type p53 (14% vs. 60.5%, p = 0.009). On the other hand, patients treated with combined treatment had a longer disease-free-time (5-years in 71.2% vs. 37.9%, p < 0.05, Figure 2A), cancer-specific-survival (5-yrs survival 71.6% vs. 34.2%, p = 0.01, Figure 2B), and overall survival (5-yrs 57.6% vs. 30.8%, p < 0.05, Figure 2C). There was no difference in the treatment modalities between the different age-groups or basic diseases. In groups with different treatment modalities the patient outcome varied: The 5-year disease-specific survival was 75.2% in the group treated with the preoperative radiotherapy, 69.9% with postoperative radiotherapy and only 40% in the group with surgery alone. This difference between the treatment groups almost reached statistical significance (p = 0.05).

## Discussion

In the present study, we have evaluated the prognostic value of *TP53 *gene aberrations in a series of 46 patients with primary head and neck squamous cell carcinoma. Analysis of *TP53 *mutations is being studied as a clinical marker [27]. When correlating results from mutation analysis to the outcome in HNSCC, so far restricted to exons 5-8 or 5-9, contradicting results have been published (for reviews see [28,29]). We found that tumours containing *TP53 *mutations in DNA-binding surface regions (L2, L3 + LSH) are more aggressive than tumours with mutations outside of those regions. Interestingly, patients affected by tumours with *TP53 *mutations in L3 and LSH have poorer survival, and in case of tumours with *TP53 *mutations in domains L2, L3 + LSH there were more relapses and shorter disease free time than with other tumours implicating a more aggressive phenotype. Similar findings have already been reported in gastric cancer [30], and colorectal carcinoma [31]. Bazan and co-workers (2005) reported that in sporadic colorectal carcinoma tumours with *TP53 *mutations in L3 domain are associated with a worse prognosis (as judged by disease free time only) than other tumours [31]. Furthermore, several studies have revealed a strong association between the mutations in the L2/L3 loop and a shorter survival or poorer response to treatment in breast cancer [32,33] and in oesophageal cancer [34]. Oral squamous cell cancers with *TP53 *mutations in DNA-binding surface regions (L2, L3 and the LSH motif) and conserved regions (II-IV) were also associated with a significantly poorer prognosis than tumours with mutations outside of those regions [35]. In a large multi-centre study of squamous cell head and neck cancer, Poeta and co-workers (2007) found that mutations affecting the protein structure and DNA-binding capacity of *TP53 *were associated with shortened disease-specific and overall survival [36]. In our study, most (78%) patients that had *TP53 *mutations in L2, L3 and LSH motifs died of cancer. Only 42% of those patients with a wild type *TP53 *or 12.5% in those with *TP53 *mutations outside the L2/L3/LSH domain died of cancer. Interestingly, the patients with mutations outside the DNA-binding domains of p53 had a better survival than the patients with a wild-type p53. The association between *TP53 *mutation types and outcome presented here is also in agreement with the largest study on the *TP53 *mutation types and association with clinical phenotypes carried out in 630 patients screened for mutations in breast cancer and published by Alsner and co-workers (2008) [37].

When we compared the disease-free survival, disease-specific or overall survival in HNSCC patients with and without *TP53 *mutations, no difference was seen between the two groups. Similarly, some researchers have failed to find such a correlation [38,39]. On the other hand, positive association between the *TP53 *mutations and poor prognosis have been published [36,40,41]. Patients with *TP53 *mutations have been shown to have a significantly shorter survival time than those without any *TP53 *mutations [42]. Also the loco-regional control rate and disease-free survival rate have been shown to be inferior in the patient group with a *TP53 *mutation when compared to the patients with a wild-type *TP53 *[43]. We show here that *TP53 *mutations in exons 6 and 8 correlate with poorer overall survival. Russo and co-workers (2006) have noticed that *TP53 *mutations within exons 5 and 8 are strong prognostic indicators of both disease recurrence and survival in patients with locally advanced laryngeal squamous cell carcinoma [41]. Similarly, Huang and co-workers (1998) have studied 204 cases of non-small cell lung carcinoma, and patients with mutations in exons 7 and 8 have a significantly shorter survival compared with patients with other mutations or no mutation [44]. Our findings together with the published data suggest that the nature and location of the mutation are connected to tumour aggressiveness and prognosis and it is not enough to analyze merely the presence or absence of the *TP53 *mutations. Thus, the usefulness of *TP53 *mutations in human tumours for clinical purposes requires a much more detailed analysis.

We found here that patients with *TP53 *mutations in L2, L3 or LSH motif received significantly less often combined treatment (curative intent) and had significantly lower disease-free time and cancer specific-survival. Therefore, specific *TP53 *mutations present in L2, L3 and LSH motifs could lead to inherently more aggressive tumours than mutations outside of these regions. The age of the patients or other diseases did not affect the treatment modalities. Mechanism of action of a mutant p53 appears to be complex [45,46]. Many studies have shown that although mutant p53 proteins often lose the ability to activate the expression of genes that are responsive to wild-type p53, they may not be inert when it comes to the regulation of transcription [16]. Indeed, a mutant p53 can acquire the ability to both positively and negatively regulate gene expression, which clearly contributes to some of the pro-tumorigenic functions of a mutant p53, such as enhanced survival and resistance to cancer therapy [16,47].

There have been a limited number of inconclusive studies on *TP53 *mutations in patients treated with primary radiotherapy (for a review, see [48]). We evaluated the relationship between *TP53 *mutations and radiosensitivity in 36 patients with HNSCC who underwent primary radiotherapy. Tumours containing *TP53 *mutations in DNA-binding surface region had significantly poorer responses to radiation than other tumours. Eriksen and co-workers (2005) have investigated the importance of *TP53 *mutations for the overall treatment time of radiotherapy in HNSCC patients [39]. They noticed that patients with carcinomas containing a wild-type p53 did not benefit as much as the patients with a mutated *TP53 *from an increase in the number of weekly fractions (i.e. a reduction in the overall treatment time), as judged by local control at T-site, disease-specific or crude survival [39]. This may be due to the decreased ability of some mutant p53 proteins to initiate apoptosis [49]. Consequently, there may be less delay in G1 resulting in increased progress through the cell cycle and increased uncontrolled proliferation [39]. Moreover, Alsner and co-workers (2001b) noticed that majority of head and neck of tumours with a mutated *TP53 *had a tumour potential doubling time below that in wild type-tumours [50]. Carcinomas with a mutated *TP53 *had higher cell proliferation potential compared to wild type-tumours. *TP53 *mutation status may thus identify patients where the dominating factor associated with outcome is an increased intrinsic radioresistance.

## Conclusions

In conclusion, this study demonstrates that the clinical outcome for head and neck cancer patients is associated with the type of *TP53 *mutations. Mutations present in DNA-binding regions were associated with poorer prognosis and clinical response to radiotherapy. Especially, *TP53 *mutations in L2 and L3 + LSH motif may be usable as a marker for selecting the treatment option and predicting prognosis among HNSCC. Predictive factors become increasingly important, in particular because new treatment modalities e.g. radiotherapy combined with EGFR generally yield more treatment-related toxicity. However, due to the small number of patients in this study further confirmation is required before reliable clinical application is possible. Studies are needed to evaluate whether specific *TP53 *mutations influence prognosis in patients treated with conventional chemoradiotherapy or radiotherapy combined with anti-EGFR treatment.

## Competing interests

The authors declare that they have no competing interests.

## Authors' contributions

JKP carried out mutation analysis, and outcome data and wrote the paper with T-TH. KHV took part in designing and supervising the study, took part in the mutation analysis, and commented and edited the manuscript. HMH carried out immunohistochemical analysis and statistical analysis, and commented the manuscript. PP provided pathology expertise, carried out immunohistological analysis and commented the manuscript. RB helped with statistical analysis and corrected statistical data. T-TH took part in designing the study, was responsible for identifying the patients, designed and supervised the study, wrote the manuscript with JKP. All authors approved the final manuscript.
